# Green Synthesis of Silver Nanoparticles Using *Euphorbia wallichii* Leaf Extract: Its Antibacterial Action against Citrus Canker Causal Agent and Antioxidant Potential

**DOI:** 10.3390/molecules27113525

**Published:** 2022-05-30

**Authors:** Muhammad Arif, Rahim Ullah, Maaz Ahmad, Ahmad Ali, Zahid Ullah, Mohammad Ali, Fakhria A. Al-Joufi, Muhammad Zahoor, Hassan Sher

**Affiliations:** 1 Centre for Plant Sciences and Biodiversity, University of Swat, Charbagh 19120, Khyber Pakhtunkhwa, Pakistan; arifshangla5@gmail.com (M.A.); ru22867@gmail.com (R.U.); maazahmad25@yahoo.com (M.A.); zahidmatta@gmail.com (Z.U.); hassan.botany@gmail.com (H.S.); 2 Centre for Biotechnology and Microbiology, University of Swat, Charbagh 19120, Khyber Pakhtunkhwa; Pakistan; alimoh@uswat.edu.pk; 3 Department of Pharmacology, College of Pharmacy, Jouf University, Skaka 72341, Aljouf, Saudi Arabia; faaljoufi@ju.edu.sa; 4 Department of Biochemistry, University of Malakand, Chakdara Dir Lower 18800, Khyber Pakhtunkhwa, Pakistan

**Keywords:** *Euphorbia wallichii*, phytochemicals, antioxidant, silver nanoparticles, *Xanthomonas axonopodis*

## Abstract

Biologically synthesized silver nanoparticles are emerging as attractive alternatives to chemical pesticides due to the ease of their synthesis, safety and antimicrobial activities in lower possible concentrations. In the present study, we have synthesized silver nanoparticles (AgNPs) using the aqueous extract of the medicinal plant *Euphorbia wallichii* and tested them against the plant pathogenic bacterium *Xanthomonas axonopodis*, the causative agent of citrus canker, via an in vitro experiment. The synthesized silver nanoparticles were characterized by techniques such as UV-Vis spectroscopy, Fourier transform infrared spectroscopy, energy-dispersive X-ray spectroscopy, X-ray diffraction analysis and transmission electron microscopy. Moreover, the plant species were investigated for phenolics, flavonoids and antioxidant activity. The antioxidant potential of the extract was determined against a DPPH radical. The extract was also evaluated for phenolic compounds using the HPLC technique. The results confirmed the synthesis of centered cubic, spherical-shaped and crystalline nanoparticles by employing standard characterization techniques. A qualitative and quantitative phytochemical analysis revealed the presence of phenolics (41.52 mg GAE/g), flavonoids (14.2 mg QE/g) and other metabolites of medicinal importance. Different concentrations (1000 µg/mL to 15.62 µg/mL—2 fold dilutions) of AgNPs and plant extract (PE) alone, and both in combination (AgNPs-PE), exhibited a differential inhibition of *X. axanopodis* in a high throughput antibacterial assay. Overall, AgNPs-PE was superior in terms of displaying significant antibacterial activity, followed by AgNPs alone. An appreciable antioxidant potential was recorded as well. The observed antibacterial and antioxidant potential may be attributed to eight phenolic compounds identified in the extract. The *Euphorbia wallichii* leaf-extract-induced synthesized AgNPs exhibited strong antibacterial activity against *X. axanopodis*, which could be exploited as effective alternative preparations against citrus canker in planta in a controlled environment. In addition, as a good source of phenolic compounds, the plant could be further exploited for potent antioxidants.

## 1. Introduction

The management of plant pathogenic diseases is of immense importance, especially in the context of economically important plants across the world [1]. These diseases result in a reduced production of valuable crops, which leads to worldwide economic losses [2,3]. For instance, *Xanthomonas axonopodis* is the causal agent of citrus canker and bacterial leaf spots in many plant species of economic importance [4]. The citrus canker disease occurs in various species of citrus and some citrus relatives, including lemon, grapefruit and orange, etc. [5]. The disease is of concern because it has no cure, and most of the control strategies are focused on restraining the dissemination of its causative agent [6]. It is a very expensive disease and millions of dollars are spent worldwide for its prevention and control [5,7]. The symptoms include leaf spotting, fruit rind-blemishing, premature fruit development and dieback, which cause extensive damage to citrus [7,8]. It is considered as one of the major threats to the citrus industry worldwide [9]. Technologies such as biological control using plant extracts [10] and the applications of nanoparticles [11,12] could be effectively used to control plant disease.

*Euphorbia* is among the largest genera of flowering plants, with c. 2000 accepted species, making it the third largest genus of angiosperm [13,14]. Many species of *Euphorbia* possess medicinal properties and have been used traditionally by native communities for the treatment of various diseases in indigenous societies [15,16,17]. *Euphorbia wallichii* Hook. f., commonly known as the Himalayan or Wallich Spurge, is native to the Himalayas from Pakistan in the north and extends eastward to Yunnan, China, between elevations of 2200 and 4000 m above sea level. It is a perennial robust, patchy-form herb, with woody rootstock, reaching up to 100 cm height, with cyathia subtended by yellowish ovate bracts and bearing a tricarpellary ovary. *Euphorbia wallichii* contains important secondary metabolites, including diterpenoids and triterpenoids [18]. Therefore, it has been used as folk medicine by different communities for the treatment of skin diseases and edema [19,20]. The methanolic extract of *E. wallichii* was previously used for the synthesis of silver nanoparticles, which has shown a significant inhibition of the selected pathogens [21]. Similarly, the ethanolic extract of *E. wallichii* rhizome was used as a bio-reductant for the synthesis of silver nanoparticles. The prepared nanoparticles revealed efficient biological activities and were found to be useful against infectious and oxidative stress disorders [22].

The plant-based synthesis of nanoparticles is preferred due to the presence of important biomolecules that may act as reducing and capping agents to synthesize nanoparticles with desired morphological properties [23,24]. Medicinal plants are of special concern since they control the size and shape of nanoparticles by providing capping layers to nanoparticles [25,26]. Among metals, silver is the most important source for the synthesis of nanoparticles due to its strong antifungal and antibacterial activities [27]. Silver nanoparticles have been previously investigated for their applications in medicine, diagnostics, cosmetics and food processing industries [28,29]. Moreover, previous reports showed that the growth of plant pathogenic bacteria and fungi has been inhibited by biologically synthesized silver nanoparticles [30,31].

To the best of our knowledge, there is no published report on AgNPs synthesis through a greener route using leaf extract of the medicinal plant *Euphorbia wallichii* as a reducing agent. Therefore, as a novel study, this work was designed to synthesize stable silver nanoparticles using aqueous leaf extract of *E. wallichii*, and to evaluate antioxidant potential and differential antimicrobial activities of the synthesized nanoparticles against the *X. axonopodis* bacterial strain. The extract was also subjected to phytochemical screening by using standard tests and the HPLC technique.

## 2. Materials and Methods

### 2.1. Extract Preparation for Phytochemical Screening

Healthy plants of *Euphorbia wallichii* were collected from the moist temperate coniferous forest in Jarugo waterfall, Biha valley, 35 km northwest of Matta Swat, Pakistan. The leaves were washed and then shade-dried at room temperature and were homogenized into fine powder by using mortar and pestle. For the preparation of extract, approximately 50 g leaf powder was added to 300 mL of distilled water in a 500 mL Erlenmeyer flask and the solution was incubated at 28 °C for 24 h. The solution was then filtered using Whatman filter paper (grade 1, pore size 11 µm) to yield a clear liquid, which was then evaporated, and the crude extract was obtained.

### 2.2. Qualitative Analysis

The qualitative screening of the leaf extract of *E. wallichii* was carried out following the protocols of Ajayi et al. [32] with minor modifications.

#### 2.2.1. Test for Terpenoids

To investigate terpenoid contents, approximately 1.0 mg crude extract was boiled in 5 mL distilled water. The solution was then filtered and mixed with 2 mL chloroform and 3 mL concentrated sulfuric acid. The appearance of reddish-brown color indicated the presence of terpenoids.

#### 2.2.2. Test for Glycosides

To investigate the glycoside contents, approximately 5 mg crude extract was heated in distilled water at 60 °C for 15 min. The prepared solution was filtered and 5 mL of filtered extract was mixed with 2 mL of glacial acetic acid and 1 mL of concentrated sulfuric acid. This was followed by the dropwise addition of ferric chloride. The appearance of blue ring at the bottom of the flask indicated the presence of glycosides.

#### 2.2.3. Test for Tannins

To investigate tannins, approximately 2 mg crude extract was taken in 20 mL distilled water and placed on hot water bath for 5 min. The solution was filtered and 1 mL of the filtrate was taken in a vial, and ferric chloride was added dropwise to it. The appearance of brownish color indicated the presence of tannins.

#### 2.2.4. Test for Flavonoids

To investigate flavonoid contents, approximately 1 mg of crude extract was taken in 10 mL distilled water and kept on hot water bath for 5 min. The solution was filtered and 1 mL of the filtrate was taken in a vial followed by the addition of sodium hydroxide (20%) dropwise. The appearance of yellow color confirmed the presence of flavonoids.

### 2.3. Quantitative Screening

#### 2.3.1. Total Phenolic Contents (TPCs)

The TPCs were determined following standard procedure of Shirazi et al. [33] with minor modifications. Briefly, 5 mg crude extract was taken in 10 mL distilled water and placed on hot water bath for 30 min. The solution was then filtered and 100 µL extract was mixed with 500 µL distilled water in a vial. Further, 100 µL of Folin-Ciocalteu reagent and 1000 µL of sodium carbonate (7%) were added to the solution, which was kept in the dark for 90 min at room temperature. The absorbance of the resultant blue-colored solution was recorded at 760 nm using UV-Vis dual beam spectrophotometer. For TPC quantification, a calibration curve of standard gallic acid was used and was expressed as mg GAE/g (gallic acid equivalent/gram) of dry sample.

#### 2.3.2. Total Flavonoid Contents Test (TFCs)

The TFC was determined following protocol of Kim et al. [34] with minor modifications. Approximately 100 µL extract was mixed with 500 µL distilled water and 100 µL NaNO_3_ (5%). Then, 150 µL of AlCl_3_ (10%) and 200 µL of sodium hydroxide (1M) were added to it. After 5 min, the absorbance was recorded at 510 nm using UV-Vis double beam spectrophotometer. A standard quercetin curve was used for TFC quantification and was expressed as mg QE/g (quercetin equivalent/gram) of dry sample.

#### 2.3.3. Antioxidant Assay

DPPH antioxidant assay of the leaf was accomplished as per the standard method of Cho et al., 2002 [35], with certain modifications. DPPH solution was prepared and kept in the dark at room temperature for 24 h. Further, sample solution was prepared by dissolving 5 mg crude extract in 5 mL methanol (90%). The obtained solution was diluted to 5 different concentrations, i.e., 62.5, 125, 250, 500 and 1000 µg/mL. Approximately 1 mL of each solution was mixed properly with 2 mL DPPH and the mixtures were kept in the dark for 30 min at room temperature. Absorbance of each mixture was checked at 517 nm. Standard ascorbic acid was used for quantification and percent activity was determined by the following formula:

(1) $$\mathrm{Percent}\;\mathrm{inhibition}=\frac{\mathrm{Absorbance}\;\mathrm{of}\;\mathrm{control}-\mathrm{Absorbance}\;\mathrm{of}\;\mathrm{sample}}{\mathrm{Absorbance}\;\mathrm{of}\;\mathrm{control}}\times 100$$

#### 2.3.4. HPLC-UV Profiling for the Detection of Phenolic Compounds

Detection of phenolic compounds in the leaf extract was performed using HPLC Agilent 1260 system equipped with UV detector, degasser and autosampler [36]. Approximately 1 g dried powdered sample was dissolved in 50% methanol (20 mL, *v*/*v* water/methanol) and kept on hot water bath at 50 °C for 1 h. After double filtration, the solution was poured into HPLC vials for the determination of phenolic compounds. ZORBAX Eclipse C18 (4.6 × 250 mm, 5 Micron) column was used for separation of components. Sample was eluted with a gradient system comprising solvent A: methanol, acetic acid, deionized water, 10:2:88, *v*/*v*, and solvent B: methanol, acetic acid, deionized water, 90:2:8, *v*/*v*. The gradient program was started with 100% A, then 85% A and 15% B at 5 min, 50% each of A and B at 20 min, 30% A and 70% B at 25 min and 100% B from 30 to 40 min, with a flow rate of 1 mL/min. The chromatograms were recorded at room temperature at 320 nm. The identification of bioactive compounds was carried out by comparing the retention times of samples peaks with that of standards.

#### 2.3.5. Biosynthesis of Silver Nanoparticles

For the biosynthesis of silver nanoparticles, 1.25 mg/mL plant extract was mixed properly with 4 mM silver nitrate in different volumetric ratios (1:9 to 9:1) as per standard protocols [37,38]. The prepared mixtures were exposed to sunlight for 15 min to observe the color change. The mixtures were incubated for 24 h following exposure to sunlight. After spectroscopic analysis, mixture with appropriate optical characteristic was selected for centrifugation and washing. For separation of silver nanoparticles, the prepared solutions were centrifuged at 14,000 rpm for 15 min. The supernatants were discarded and the settled materials were dissolved in deionized water and were centrifuged again to remove unreacted reactants and impurities. This process was repeated three times to obtain pure and washed silver nanoparticles (AgNPs). Following the final step of washing, the supernatants were pipetted out and the Eppendorf tubes (1.5 mL) with AgNPs pellets were kept open for 24 h at room temperature to obtain dried AgNPs. The dried AgNPs were later characterized through various physical techniques. To prepare plant-extract-coated silver nanoparticles (AgNPs-PE), the dried AgNPs were re-immersed in 1.25 mg/mL plant extract and air dried.

### 2.4. Characterization of Silver Nanoparticles

Different physical techniques were used for the characterization of biosynthesized silver nanoparticles, which were as follows:

MultiskanTM Sky Microplate Spectrophotometer (MAN0018930) was used for UV-visible spectroscopy of biosynthesized silver nanoparticles. The surface plasma resonance was recorded at the spectral range of 300–600 nm to find out the typical peak for silver.

Thermo-Nicolet 6700 FTIR Spectrometer (Madison, WI, USA) ranging from 4000 to 400 cm^−1^ was used in ATR reflection mode using a Ge crystal to detect functional groups responsible for the formation of stable nanoparticles. The observed peaks of FTIR analysis was compared with IR spectrum table to identify the specific functional group.

JEOL JEM-101 system was used for transmission electron microscopy of the prepared nanoparticles. The exploration of size and shape morphology was recorded using different magnification lens. Moreover, selected area electron diffraction (SAED) was performed to determine the crystallographic structure.

JSM5910, JEOL, Japan, scanning electron microscope equipped with energy dispersive X-ray system was used for EDX analysis to determine the elemental composition of the particles.

JDX-3432, JEOL, Japan was used for XRD pattern analysis of the synthesized nanoparticles, following the protocol of Asghar et al. [39]. The average size of crystallite was measured using Debye–Scherrer equation, which is calculated by D = k λ/βcosθ,

Where D is the average size, k is constant factor (0.9), λ is wavelength of X-ray, β is full width at half maximum of the peak in radians and θ is angle diffraction.

### 2.5. Antibacterial Assay against Xanthomonas axonopodis

Biosynthesized AgNPs (AgNPs and AgNPs-PE) were investigated for their antibacterial efficiency against *Xanthomonas axonopodis* using microtiter plate assay [40]. Briefly, the antibacterial activity was performed by using 96-well microtiter plate with varying concentrations (1000 µg/mL, 500 µg/mL, 250 µg/mL, 125 µg/mL, 62.5 µg/mL, 31.25 µg/mL, 15.62 µg/mL) of washed silver nanoparticles (AgNPs), plant extract (PE) and plant-extract-coated silver nanoparticles (AgNPs-PE). Distilled water was used to prepare 1000 µg/mL of dried AgNPs, AgNPs-PE and plant extract. Lower concentrations of each treatment were prepared with distilled water by 2-fold serial dilution method. Pure culture of *X. axonopodis* was obtained from Department of Plant Pathology, University of Peshawar. The culture was refreshed on nutrient agar 2–3 times by incubation at 28 °C for 36 h each time. Following growth on nutrient agar, cultures were established in nutrient broth and incubated at 28 °C overnight in shaker incubator at 200 rpm. For antibacterial assay, the OD of bacterial suspensions was adjusted as OD (600) = 1. To make a total volume of 300 µL in each well of microtiter plate, 150 µL of bacterial suspension was mixed with 150 µL of each treatment concentration and the plates were placed in shaking incubator at 28 °C and 200 rpm. Control treatment used in the experiment consisted of bacterial cells without nanoparticles or plant extract. Each treatment was replicated three times and the optical density was recorded at 600 nm at 0 h and 24 h time points. The growth inhibition pattern of the bacterial cells was calculated by using the formula:

Antibacterial activity (%) = Control-Treatment/Control × 100(2)

## 3. Results

### 3.1. Phytochemical Screening

**Figure 1:**
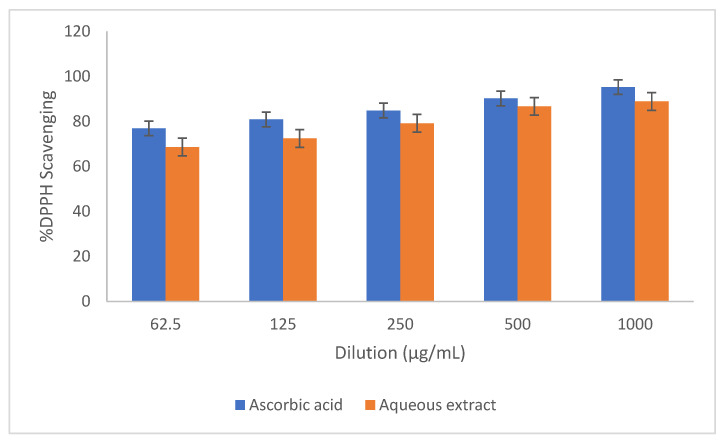
Free radical scavenging effects of aqueous extract of *E. wallichii* (IC_50_ of ascorbic acid = 10 µg/mL, IC_50_ of aqueous extract = 32 µg/mL).

**Figure 2:**
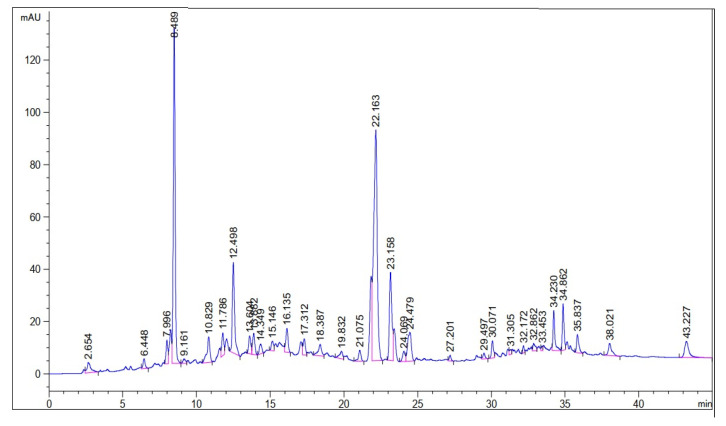
HPLC phenolic profile chromatogram of *E. wallichii*.

A qualitative analysis of the methanolic extract of *E. wallichii* showed the presence of terpenoids glycosides, tannins and flavonoids in the aqueous extract (Table 1). Total phenolic and flavonoid contents are also given in Table 1. Moreover, the DPPH antioxidant activity of the methanolic extract of *E. wallichii* revealed the highest percent activity for the dilution of 1000 µg/mL. The results regarding DPPH activity were compared with standard ascorbic acid and the obtained data are presented in Figure 1. The extract contained antioxidant agents in the form of phytochemical, and scavenged the DPPH radical effectively, with an IC_50_ of 32 µg/mL in comparison to the standard (IC_50_ of ascorbic acid = 10 µg/mL). The HPLC profiling of the *E. wallichii* leaf extract identified nine bioactive compounds (Figure 2 and Table 2). The most prominent possible bioactive phenolic compounds identified were mandelic acid and morin. The observed antioxidant and antimicrobial potential may be attributed to the compounds identified.

The observed biological potential may be due to the presence of these compounds. However, at this stage, it is too early to claim this, and further studies are needed.

### 3.2. Characterization of Biosynthesized Silver Nanoparticles (AgNPs)

**Figure 3:**
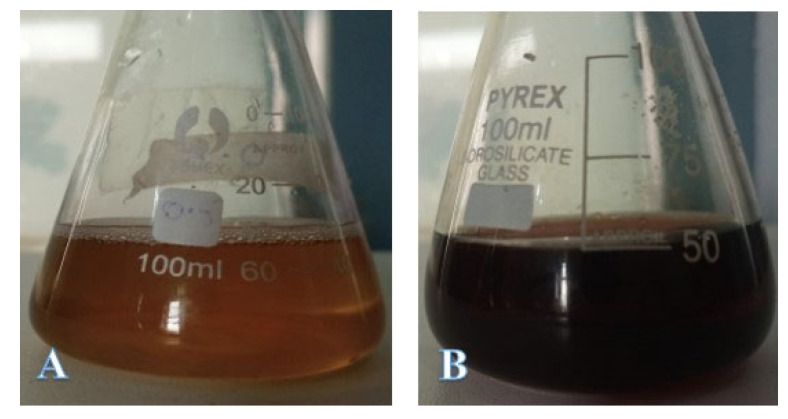
Photographs of leaf extract *of Euphorbia wallichii* (**A**) and reaction mixture containing AgNO_3_ solution (4 mM) and *E. wallichii* leaf extract (**B**).

**Figure 4:**
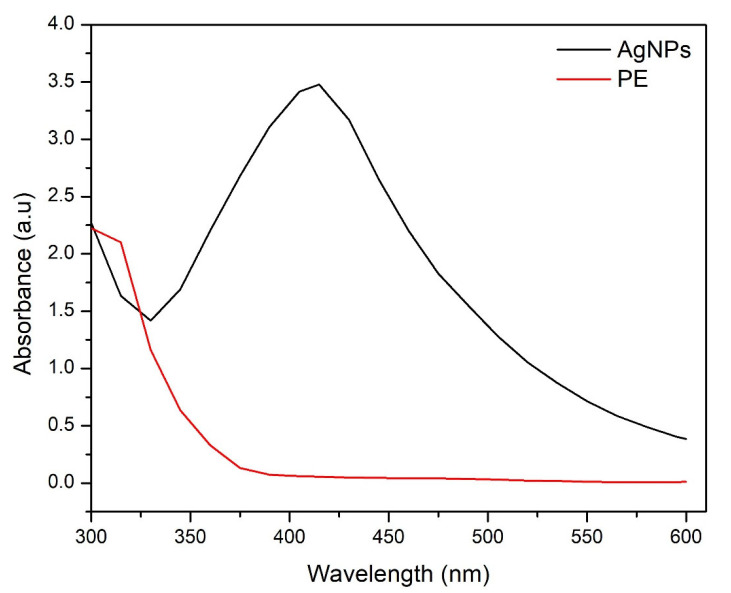
Optical (UV-visible) graph of washed silver nanoparticles (AgNPs) after 24 h of mixing the reactants and plant extract (PE).

After mixing the plant extract and silver nitrate in different proportions, the reaction mixtures turned dark brown when exposed to direct sunlight, which is a common characteristic of the synthesized silver nanoparticles in aqueous form (Figure 3b). The UV-visible absorption spectra of the mixtures of plant extract and silver nitrate in different volumetric ratios showed an increased absorbance between 400–500 nm. However, the mixture with a 1:1 volumetric ratio exhibited the best spectral characteristics, and a narrow peak was observed at 415 nm after 24 h of incubation (Figure 4). The band gap energy calculated from the UV-visible data was found to be 1.79 eV.

**Figure 5:**
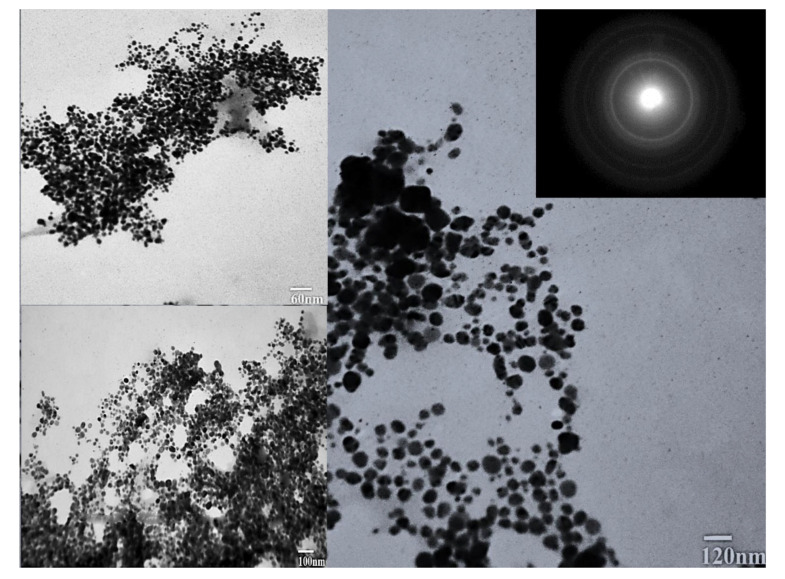
TEM micrographs and SAED pattern of the biosynthesized silver nanoparticles (AgNPs).

**Figure 6:**
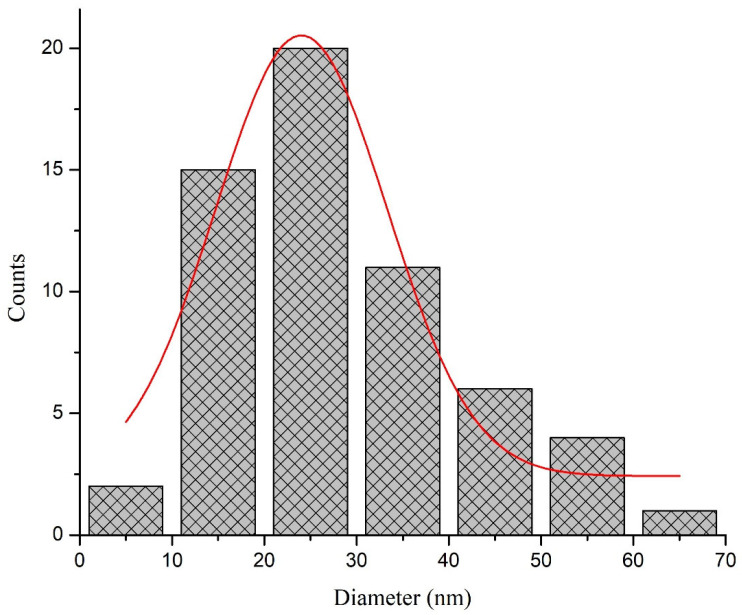
Size distribution histogram of the biosynthesized silver nanoparticles (AgNPs).

The TEM micrographs of biosynthesized silver nanoparticles showed round and spherical-shaped silver nanoparticles (Figure 5). The size of the synthesized nanoparticles ranged from 20 to 60 nm, with an average size of 24 nm. The TEM images showed aggregates of particles; however, these were not in direct contact. Moreover, the SAED pattern records Bragg reflection rings equivalent to the crystalline nature of silver nanoparticles. The size distribution histogram is presented in Figure 6.

**Figure 7:**
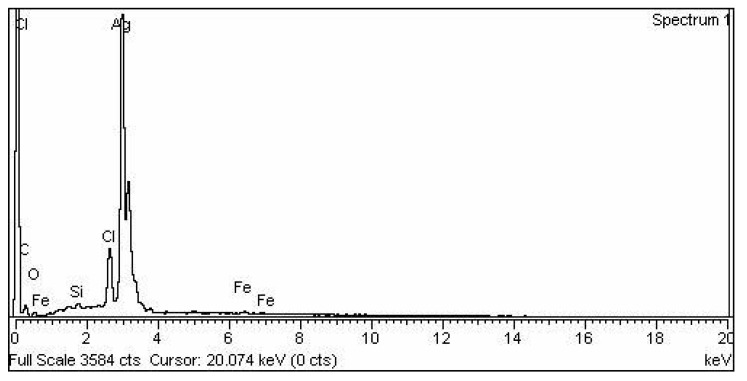
EDX spectra of the biosynthesized silver nanoparticles (AgNPs).

The graphical results for the energy dispersive X-ray (EDX) are presented in Figure 7. The elemental composition showed the highest amount, with a strong peak of the silver element in the EDX spectra. The specific signal between 3 to 4 keV was observed, which confirmed the presence of silver metal.

**Figure 8:**
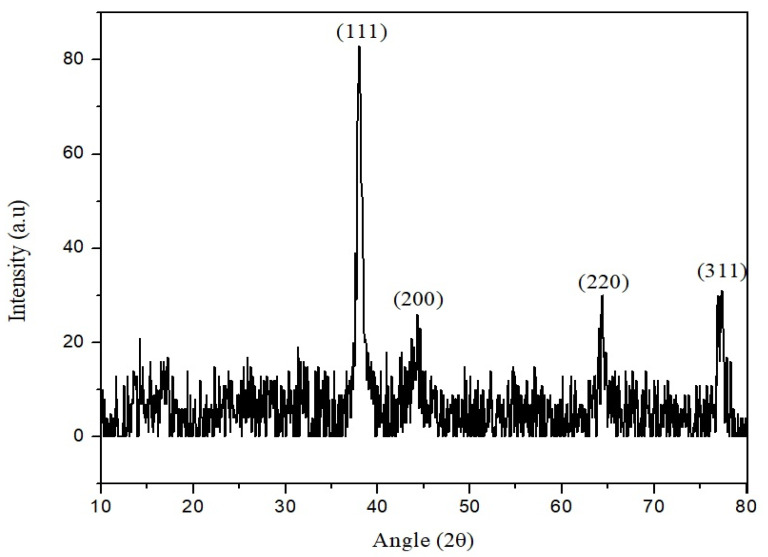
XRD pattern of the biosynthesized silver nanoparticle (AgNPs).

The crystalline nature of AgNPs was confirmed through XRD analysis, where 2 θ values were taken on the *X*-axis and corresponding intensities were taken on the *Y*-axis (Figure 8). The crystalline nature of the synthesized AgNPs was patterned using XRD analysis in the range of 10° to 80° at a 2θ diffraction angle. The four diffraction peaks at ~38°, ~45°, ~65° and ~78° were attributed to the crystalline planes 111, 200, 220 and 311, respectively. The XRD study showed the cubic crystal shape with an average crystallite size of 32 nm (according to the Debye–Scherrer equation) of the synthesized silver nanoparticles.

**Figure 9:**
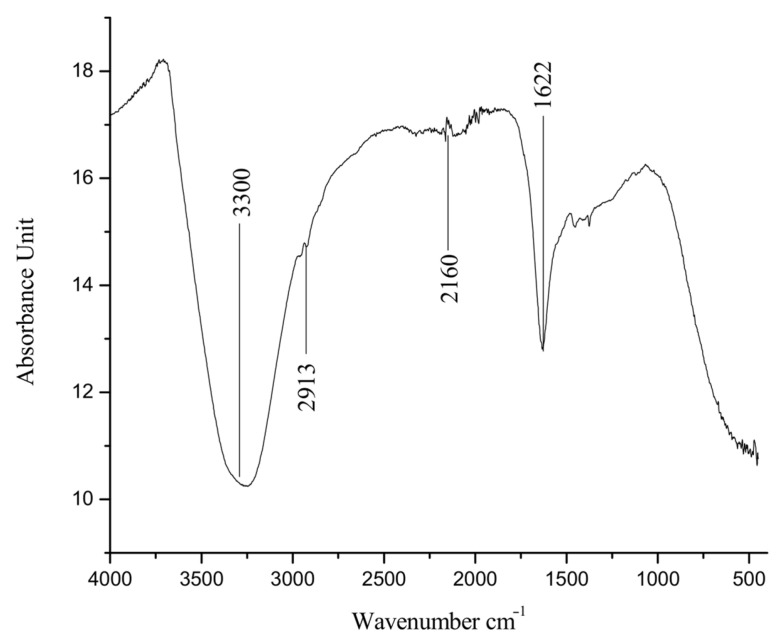
FTIR spectrum of the biosynthesized silver nanoparticles (AgNPs).

FTIR results of the AgNPs are given in Figure 9, which show the presence of different functional groups. FTIR spectra found that a different peak at 3300 cm^−1^ is associated with OH stretching corresponding to alcohols, 2913 for C-H stretching of alkene, 2160 for C≡C stretching of alkyne and 1622 for C=C stretching of alkene. These functional groups were probably the capping layer of the plant secondary constituent, which is responsible for stable nanoparticle formation.

### 3.3. Antibacterial Activity of the Biosynthesized Nanoparticles

**Figure 10:**
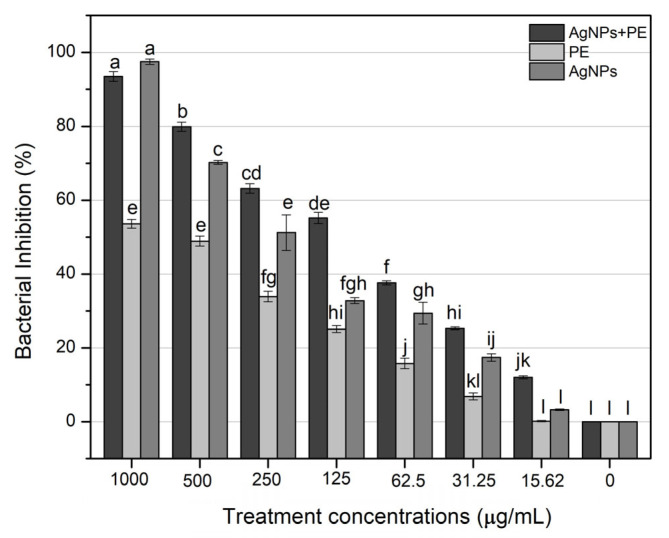
Inhibition of *X. axonopodis* in response to different concentrations (1000 µg/mL, 500 µg/mL, 250 µg/mL, 125 µg/mL, 62.5 µg/mL, 31.25 µg/mL, 15.62 µg/mL) of washed silver nanoparticles (AgNPs), plant extract-coated silver nanoparticles (AgNPs-PE) and plant extract (PE). Bars with zero concentration represent control. Bars with the same letters are not significantly different. Different concentrations of each treatment were analyzed by Tukey’s HSD separately.

The AgNPs (1000 µg/mL) and AgNPs-PE (1000 µg/mL) strongly inhibited the growth of *X. axonopodis*, while PE showed an optimum inhibition in comparison to the control (water) treatment. Our finding revealed that the highest inhibition was 98% by AgNPs (1000 µg/mL) and 93% by AgNPs-PE (1000 µg/mL), which were significantly comparable. However, the plant extract (1000 µg/mL) alone inhibited the growth by 53.6%. No inhibition in the cell growth of *X. axanopodis* was observed by the control treatment (Figure 10).

## 4. Discussion

Medicinal plants are of special concern for the efficient synthesis of silver nanoparticles, since they control the size and shape of the particles [41,42]. Plants are easily available and can be utilized in limited quantities for the synthesis process [43]. Plants contain important secondary metabolites that provide stability to nanoparticles [44]. Moreover, green synthesis does not use any toxic chemicals and offers a safe and bio-friendly route for the large-scale production of nanoparticles [45].

The reducing potential of different classes of plant secondary metabolites to synthesize nanoparticles has been excellently reviewed [46,47]. Biomolecules with various structural classes, such as phenolics, flavonoids, terpenoids, saponins, alkaloids, etc., that are present in plant extracts may be involved as reducing and stabilizing agents in nanoparticles synthesis; however, complexity regarding the exact mechanism of synthesis exists [48,49]. Notwithstanding, the complex nature of plant extracts, the synergism of metabolites in reducing metal ions and differential phytochemical profiles of plant species may be the factors that contribute to uncertainty regarding a generalized mechanism. A recent study conducted by Pradeep et al. [48] demonstrated that compounds such as kaempferol-3-glucoside, quercetin and quercetin-3-glucoside are able to reduce silver ion sizes. The same study showed that metabolites with enol groups reduce metal ions to nanoparticles due to their antioxidant potential and metabolites with methoxy groups act as capping agents. In another study, quercetin in *Ocimum sanctum* leaf extract was considered as the main compound used to reduce silver ions to silver nanoparticles [50]. However, the dual nature of secondary metabolites, such as terpenes, as reducing agents and capping agents has also been demonstrated [51]. In the current study, phytochemical investigations were conducted to determine the quantity of major bioactive compounds as possible reducing agents of silver ions, and the presence of other classes of metabolites was investigated for their possible capping properties of silver nanoparticles. The results showed a rich profile of bioactive compounds, predominantly epigallocatechin, rutin, quercetin and morin, which may be more likely involved as the major reducing compounds of silver ions.

In this study, physical characterization techniques revealed that most of the synthesized AgNPs were round and spherical-shaped, in the size range of 20 nm to 60 nm and crystalline in nature. Moreover, alkene, alkyne and alcohol groups were found to be associated with AgNPs, suggesting the involvement of these plant-extract-associated functional groups in reducing the potential of ionic silver to a particulate form. Consistent with our results, several studies focused on the green synthesis of AgNPs, and their physical characteristics and functional groups as reducing agents have been reviewed recently [52]. Chakraborty et al. [53] have found alkane and alkene as the possible reducing agents of silver ions to atomic silver by using *Galphimia glauca* leaf extract. Moreover, the major energy peak corresponding to silver by EDX analysis was found to be between 3 and 4 keV, which is consistent with the findings of Ali et al. [54]. The optical band gap, characterized as the gap between the valence band and the conduction band, was found to be 1.79 eV for the synthesized AgNPs. Our results on the interrelation of UV-Vis absorption, band gap energy and the size of AgNPs are comparable with the findings of Roddu et al. [55].

We found a significant impact of the volumetric ratios of *Euphorbia wallichii* leaf extract and silver nitrate on AgNPs synthesis. Both the reactants mixed in a 1:1 *v*/*v* ratio exhibited an AgNPs sample with the desired physical characteristics, such as the size, shape and consistency of physical parameters. The differential profile of AgNPs synthesis following the mixing of plant extract and silver nitrate has previously been reported [54]. Similarly, we found that the incubation of a reactant mixture in direct sunlight stimulated the reaction substantially, as evident by the abrupt color change. Previous studies have shown the same pattern of the accelerated synthesis of AgNPs following exposure to sunlight [55,56,57].

Pathogenic bacteria cause a number of diseases of valuable crops and reduce their productivity [58]. For instance, *X. axonopodis* causes citrus canker and bacterial leaf spots in the member species of family Rosaceae [4], and lead to a compromised quality and quantity of landscape plants. Similarly, these diseases reduce the crop productivity of citrus species worldwide, leading to huge economic losses [4,59]. Chemical pesticides are traditionally used to control these plant pathogens; however, the use of synthetic chemicals is hazardous to other organisms, including humans [60]. Therefore, biosynthesized silver nanoparticles may offer alternative control formulations due to their innocuous nature to control phytopathogens efficiently [61,62]. Biosynthesized silver nanoparticles are non-toxic, cost effective and efficient against pathogenic microbes [37]. Several studies have reported the efficiency of biosynthesized silver nanoparticles against pathogenic microbes [40,42,63]. In this study, AgNPs alone and plant-extract-coated silver nanoparticles (AgNPs-PE) displayed strong antibacterial activity at lower concentrations compared to plant extract alone. Overall, dose-dependent inhibition profiles were exhibited by AgNPs, AgNPs-PE and PE. Interestingly, more than a 50% bacterial inhibition occurred in response to 250 µg/mL of AgNPs and AgNPs-PE. Moreover, significantly comparable activity was shown by 250 µg/mL and 125 µg/mL of AgNPs-PE. This indicates that lower concentrations of AgNPs could be effectively used against the tested bacterial pathogen.

The antimicrobial efficacy of different *Euphorbia* species is well documented in previous studies [38,64,65,66,67]. Previously, Li et al. [68] have reported the isolation of three antibacterial ent-abietane-type diterpenoids from the root extract *E. wallichii*. However, Jayalakshmi et al. [65] have attributed the antibacterial activity of *Euphorbia cotinifolia* to phenols and flavonoids. In the current study, higher concentrations (1000 µg/mL and 500 µg/mL) of aqueous leaf extract showed comparable antibacterial activity, which was reduced significantly in response to lower concentrations. However, the differential effects of natural antibacterial present in the plant extracts may be due to various classes of secondary metabolites, their possible synergism and the solvents used for extraction.

In this study, we investigated the phytochemical profile and the antioxidant and antibacterial activities of the leaf extract of *E. wallichii*. The qualitative phytochemical assessment showed positive results for terpenoids, glycosides, tannins and flavonoids. The obtained results were compared with the previous studies [69,70]. The TPC in aqueous and methanolic extract was found to be 41.52 and 45.35 mg GAE/g, respectively. Additionally, the highest TFC found in the sample dilution of 1000 µg/mL was 59.34 mg QE/g, and obtained results correlated with previous studies [34,71,72].

Further, the highest DPPH free radical scavenging activity shown was 91.5 by the leaf extract of *E. wallichii*. The HPLC profiling revealed nine bioactive compounds in the leaf extract of the plant. The obtained results of the DPPH antioxidant and HPLC phenolic profiling were compared with previous literature studies [36,66,71,73,74,75]. The TEM, EDX and XRD characterization of the biosynthesized silver nanoparticles showed spherical-shaped particles that were crystalline in nature. The size of the particles ranged from 20 to 60 nm, with an average size of 24 nm. Moreover, the capping layers that provides stability to the particles were observed during FTIR study. The results of the characterization correlated with the previous studies [54,71,76,77,78].

The biosynthesized silver nanoparticles (AgNPs and AgNPs-PE) revealed significant antibacterial activity against *X. axonopodis*. The in vitro microtiter plate experiment revealed a pronounced efficiency of AgNPs-PE against the tested pathogenic bacterium. The plant secondary constituents provide stability to silver metal and increase its antibacterial effects. Our results regarding the antimicrobial activity of the biosynthesized silver nanoparticles are in agreement with those previously reported [79,80,81]. Although the exact mechanism of the antimicrobial action of silver nanoparticles is unclear, several models have been proposed. AgNPs are considered to show their antimicrobial action by causing membrane leakage, protein denaturation, DNA damage and the disassembly of bacterial ribosomes [82]. Another model proposes that Ag ions are released by AgNPs that interact with sulfur proteins in the cell wall and cytoplasmic membrane, deactivate respiratory enzymes, disrupt adenosine triphosphate (ATP) synthesis and compromise DNA replication and cell reproduction by interacting with sulfur and phosphorus in the DNA [83]. In a recent study, the in vitro inhibition of *Actinomyces viscosus* and *Streptococcus mutans* was reported due to an increase in reactive oxygen species (ROS) production and leakage of nucleic acids and proteins after treatment with green synthesized AgNPs [84]. In the current study, AgNPs alone and AgNPs in combination with plant extract displayed diverse antibacterial profiles, which demonstrates the possible synergistic role of nanoparticles and organic capping agents in bacterial inhibition. Moreover, the antibacterial action of biosynthesized nanoparticles may be strongly dependent on the polydispersity of synthesized nanoparticles and the plant extract initially used for nanoparticles synthesis.

Previous research used the ethanolic and methanolic extract of *E. wallichii* for the synthesis of silver nanoparticles [21,22]. Their finding revealed the biological efficiency of the synthesis silver nanoparticles. However, methanolic and ethanolic extract may be hazardous to human health due to the presence of sufficient amounts of methanol/ethanol [85,86]. Therefore, in this study, we have reported the synthesis of silver nanoparticles using the aqueous extract of *E. wallichii*. Our results revealed the optimal synthesis of silver nanoparticles from the aqueous leaf extract of *E. wallichii*, along with its significant antibacterial activity against *X. axonopodis*.

## 5. Conclusions

In the current study, the phytochemical profile of medicinally important *E. wallichii* was evaluated and its aqueous solution was used as a reductant in the biosynthesis of silver nanoparticles. Qualitative and quantitative phytochemical results revealed the presence of substantial quantities of secondary metabolites. From the HPLC chromatogram, nine biologically active compounds were identified. The aqueous extract potently inhibited the DPPH free radical, pointing towards the antioxidant applications of *E. wallichii* which needs to be investigated further. Plant extract revealed the total phenolic content and total flavonoid contents as 41.52 mg GAE/g and 14.2 mg QE/g, respectively. The biosynthesized silver nanoparticles were found to be in the size range of 20 to 60 nm and mostly round or spherical in shape. Moreover, alkane, alkene and alcohol were found to be the major functional groups attached to AgNPs. AgNPs also exhibited potent antibacterial activity against *X. axanopodis* in the in vitro experiment. The selected plant in the form of aqueous extract and fabricated nanoparticles has tremendous biological potential. Antioxidant and antimicrobial activities of the extract and nanoparticles have been investigated in this study; the plant in these forms needs to be investigated for other biological potentials as well.

## Figures and Tables

**Table 1:** Representative phytochemical groups present, total phenolic and flavonoid contents in the leaf extract of *E. wallichii*.

Phytochemical Group	Remarks
Tannin	Present
Terpenoid	Present
Flavonoid	Present
Glycoside	Present
TPC in aqueous extract	41.52 (mg GAE/g)
TFC in aqueous extract	14.2 (mg QE/g)

**Table 2:** Phenolic compounds identified by HPLC profiling of *E. wallichii* leaf extract.

Retention Time (min)	Peak Area (mAU*s)	Proposed Identified Compound	Identification Reference
2.654	71.0622	Vitamin C	standard
8.489	1113.0945	Mandelic acid	standard
11.786	86.6307	Caffeic acid	standard
15.146	40.0379	Hydroxy benzoic acid	standard
19.832	43.8526	Chlorogenic acid	Standard
22.163	1479.6410	Morin	standard
29.497	22.6935	Quercetin	standard
35.837	75.0610	Pyrogallol	standard
38.021	75.0148	Rutin	Standard

## Data Availability

Not applicable.

## References

[1] Shuping D.S.S., Eloff J.N. (2017). The use of plants to protect plants and food against fungal pathogens: A review. Afr. J. Tradit. Complement. Altern. Med..

[2] Strange R.N., Scott P.R. (2005). Plant disease: A threat to global food security. Annu. Rev. Phytopathol..

[3] Perombelon M.C.M. (2002). Potato diseases caused by soft rot erwinias: An overview of pathogenesis. Plant Pathol..

[4] Mohammadi M., Mirzaee M.R., Rahimian H. (2001). Physiological and biochemical characteristics of Iranian strains of *Xanthomonas axonopodis* pv. citri, the causal agent of citrus bacterial canker disease. J. Phytopathol..

[5] Gottwald T.R. (2000). Citrus canker. The Plant Health Instr..

[6] Martins P.M., Wood T.K., de Souza A.A. (2021). Persister cells form in the plant pathogen xanthomonas citri subsp. citri under different stress conditions. Microorganisms.

[7] Gottwald T.R., Graham J.H., Schubert T.S. (2002). Citrus canker: The pathogen and its impact. Plant Health Prog..

[8] Das A.K. (2003). Citrus canker—A review. J. Appl. Hortic..

[9] Hameed A., Atiq M., Ahmed Z., Rajput N.A., Younas M., Rehman A., Alam M.W., Sarfaraz S., Liaqat N., Fatima K. (2022). Predicting the impact of environmental factors on citrus canker through multiple regression. PLoS ONE.

[10] Liaquat F., Qunlu L., Arif S., Haroon U., Saqib S., Zaman W., Jianxin S., Shengquan C., Li L.X., Akbar M. (2021). Isolation and characterization of pathogen causing brown rot in lemon and its control by using ecofriendly botanicals. Physiol. Mol. Plant Pathol..

[11] Saqib S., Zaman W., Ullah F., Majeed I., Ayaz A., Hussain Munis M.F. (2019). Organometallic assembling of chitosan-Iron oxide nanoparticles with their antifungal evaluation against Rhizopus oryzae. Appl. Organomet. Chem..

[12] Saqib S., Zaman W., Ayaz A., Habib S., Bahadur S., Hussain S., Muhammad S., Ullah F. (2020). Postharvest disease inhibition in fruit by synthesis and characterization of chitosan iron oxide nanoparticles. Biocataly. Agric. Biotechnol..

[13] Shi Q.W., Su X.H., Kiyota H. (2008). Chemical and pharmacological research of the plants in genus *Euphorbia*. Chem. Rev..

[14] Riina R., Peirson J.A., Geltman D.V., Molero J., Frajman B., Pahlevani A., Barres L., Morawetz J.J., Salmaki Y., Zarre S. (2013). A worldwide molecular phylogeny and classification of the leafy spurges, *Euphorbia* subgenus *Esula* (Euphorbiaceae). Taxon.

[15] Kumar S., Malhotra R., Kumar D. (2010). *Euphorbia hirta*: Its chemistry, traditional and medicinal uses, and pharmacological activities. Phcog. Rev..

[16] Pascal O.A., Bertrand A.E.V., Esaïe T., Sylvie H.A.M., Eloi A.Y. (2017). A review of the ethnomedical uses, phytochemistry and pharmacology of the *Euphorbia* genus. Pharm. Innov..

[17] Ernst M., Grace O.M., Saslis-Lagoudakis C.H., Nilsson N., Simonsen H.T., Rønsted N. (2015). Global medicinal uses of *Euphorbia* L. (Euphorbiaceae). J. Ethnopharmacol..

[18] Ali I., Naz R., Khan W.N., Gul R., Choudhary M.I. (2009). Biological screening of different root extracts of *Euphorbia wallichii*. Pak. J. Bot..

[19] Ul-Haq I., Ullah N., Bibi G., Kanwal S., Ahmad M.S., Mirza B. (2012). Antioxidant and cytotoxic activities and phytochemical analysis of *Euphorbia wallichii* root extract and its fractions. Iran. J. Pharm. Res. IJPR.

[20] Hassan A., Yaqoob U., Nawchoo I.A., Gulzar S., Mohi-Ud-Din G., Nazir S., Ashraf A. (2016). Conspectus of phytochemical constituents of *Euphorbia wallichii* Hook. f.: A review. Res. Rev. J. Bot..

[21] Phull A.R., Ali A., Ali A., Abbasi S., Zia M., Khaskheli M.H., Kamal M.A. (2020). Synthesis of Silver Nanoparticles using *Euphorbia wallichii* Extract and Assessment of their Bio-functionalities. Med. Chem..

[22] Ullah R., Ud Din S., Muhammad Z., Shah S., Jan S.A. (2018). Biological efficacy of phyto-synthetic silver nanoparticles using ethanol extract of *Euphorbia wallichii* Hook Rhizome as bio-reductant and surfactant. Trop. J. Pharm. Res..

[23] Jagadeesh B.H., Prabha T.N., Srinivasan K. (2004). Improved shelf life of bell *Capsicum* fruits by manipulation of the activities of glycosidases through heat treatment. Indian J. Plant Physiol..

[24] Collera-Zuniga O., Jimenez F.G., Gordillo R.M. (2005). Comparative study of carotenoid composition in three mexican varieties of *Capsicum annuum* L. Food Chem..

[25] Rauwel P., Küünal S., Ferdov S., Rauwel E. (2015). A review on the green synthesis of silver nanoparticles and their morphologies studied via TEM. Adv. Mater. Sci. Eng..

[26] Iravani S., Zolfaghari B. (2013). Green synthesis of silver nanoparticles using *Pinus eldarica* bark extract. Biomed. Res. Int..

[27] Ahmed S., Ahmad M., Swami B.L., Ikram S. (2016). A review on plants extract mediated synthesis of silver nanoparticles for antimicrobial applications: A green expertise. J. Adv. Res..

[28] Sharma V.K., Yngard R.A., Lin Y. (2009). Silver nanoparticles: Green synthesis and their antimicrobial activities. Adv. Colloid Interface Sci..

[29] Thorley A.J., Tetley T.D. (2013). New perspectives in nanomedicine. Pharmacol. Ther..

[30] Kim J.S., Kuk E., Yu K.N., Kim J.H., Park S.J., Lee H.J., Kim S.H., Park Y.K., Park Y.H., Hwang C.Y. (2007). Antimicrobial effects of silver nanoparticles. Nanomed. Nanotechnol. Biol. Med..

[31] Jong W.H.D., Borm P.J.A. (2008). Drug delivery and nanoparticles: Applications and hazards. Int. J. Nanomed..

[32] Ajayi I.A., Ajibade O., Oderinde R.A. (2011). Preliminary phytochemical analysis of some plant seeds. Res. J. Chem. Sci..

[33] Shirazi O.U., Khattak M.M.A.K., Shukri N.A.M., Nasyriq M.N. (2014). Determination of total phenolic, flavonoid content and free radical scavenging activities of common herbs and spices. J. Phacog. Phytochem..

[34] Kim D.O., Jeong S.W., Lee C.Y. (2003). Antioxidant capacity of phenolic phytochemicals from various cultivars of plums. Food Chem..

[35] Cho S.Y., Park J.Y., Park E.M., Choi M.S., Lee M.K., Jeon S.M., Park Y.B. (2002). Alternation of hepatic antioxidant enzyme activities and lipid profile in streptozotocin-induced diabetic rats by supplementation of dandelion water extract. Clin. Chim. Acta.

[36] Khayam S.M., Zahoor M., Shah A.B. (2019). Biological and phytochemical evaluation of cotoneaster microphyllus, *Ficus auriculata* and *Calotropis procera*. Lat. Am. J. Pharm..

[37] Castillo-Henríquez L., Alfaro-Aguilar K., Ugalde-Álvarez J., Vega-Fernández L., Montes de Oca-Vásquez G., Vega-Baudrit J.R. (2020). Green synthesis of gold and silver nanoparticles from plant extracts and their possible applications as antimicrobial agents in the agricultural area. Nanomaterials.

[38] Kemboi D., Langat M.K., Siwe-Noundou X., Krause R.W., Isaacs M.L., Tembu V.J. (2022). In vitro antibacterial and cytotoxic effects of *Euphorbia grandicornis* Blanc chemical constituents. BMC Complement. Med. Ther..

[39] Asghar M., Habib S., Zaman W., Hussain S., Ali H., Saqib S. (2020). Synthesis and characterization of microbial mediated cadmium oxide nanoparticles. Microsc. Res. Tech..

[40] Ali M., Kim B., Belfield K.D., Norman D., Brennan M., Ali G.S. (2015). Inhibition of *Phytophthora parasitica* and *P. capsici* by silver nanoparticles synthesized using aqueous extract of *Artemisia absinthium*. Phytopathology.

[41] Alam M.T., Rauf M.A., Siddiqui G.A., Owais M., Naeem A. (2018). Green synthesis of silver nanoparticles, its characterization, and chaperone-like activity in the aggregation inhibition of α-chymotrypsinogen A. Int. J. Biol. Macromol..

[42] Mata R., Nakkala J.R., Sadras S.R. (2015). Catalytic and biological activities of green silver nanoparticles synthesized from *Plumeria alba* (frangipani) flower extract. Mater. Sci. Eng. C.

[43] Basnet P., Chanu T.I., Samanta D., Chatterjee S. (2018). A review on bio-synthesized zinc oxide nanoparticles using plant extracts as reductants and stabilizing agents. J. Photochem. Photobiol. B Biol..

[44] Ahmed S., Saifullah S., Ahmad M., Swami B.L., Ikram S. (2016). Green synthesis of silver nanoparticles using *Azadirachta indica* aqueous leaf extract. J. Radiat. Res. Appl Sci..

[45] de Souza T.A.J., Souza L.R.R., Franchi L.P. (2019). Silver nanoparticles: An integrated view of green synthesis methods, transformation in the environment, and toxicity. Ecotoxicol. Environ. Saf..

[46] Mustapha T., Misni N., Ithnin N.R., Daskum A.M., Unyah N.Z. (2022). A Review on Plants and Microorganisms Mediated Synthesis of Silver Nanoparticles, Role of Plants Metabolites and Applications. Int. J. Environ. Res. Public Health.

[47] Siddiqi K.S., Husen A., Rao R.A. (2018). A review on biosynthesis of silver nanoparticles and their biocidal properties. J. Nanobiotechnol..

[48] Pradeep M., Kruszka D., Kachlicki P., Mondal D., Franklin G. (2021). Uncovering the Phytochemical Basis and the Mechanism of Plant Extract-Mediated Eco-Friendly Synthesis of Silver Nanoparticles Using Ultra-Performance Liquid Chromatography Coupled with a Photodiode Array and High-Resolution Mass Spectrometry. ACS Sustain. Chem. Eng..

[49] Marslin G., Siram K., Maqbool Q., Selvakesavan R.K., Kruszka D., Kachlicki P., Franklin G. (2018). Secondary metabolites in the green synthesis of metallic nanoparticles. Materials.

[50] Jain S., Mehata M.S. (2017). Medicinal Plant Leaf Extract and Pure Flavonoid Mediated Green Synthesis of Silver Nanoparticles and Their Enhanced Antibacterial Property. Sci. Rep..

[51] Mashwani Z., Khan M.A., Khan T., Nadhman A. (2016). Applications of plant terpenoids in the synthesis of colloidal silver nanoparticles. Adv. Colloid Interface Sci..

[52] Bamal D., Singh A., Chaudhary G., Kumar M., Singh M., Rani N., Mundlia P., Sehrawat A.R. (2021). Silver nanoparticles biosynthesis, characterization, antimicrobial activities, applications, cytotoxicity and safety issues: An updated review. Nanomaterials.

[53] Chakraborty B., Kumar R.S., Almansour A.I., Kotresha D., Rudrappa M., Pallavi S.S., Hiremath H., Perumal K., Nayaka S. (2021). Evaluation of antioxidant, antimicrobial and antiproliferative activity of silver nanoparticles derived from Galphimia glauca leaf extract. J. King Saud Univ. Sci..

[54] Ali M., Kim B., Belfield K.D., Norman D., Brennan M., Ali G.S. (2016). Green synthesis and characterization of silver nanoparticles using *Artemisia absinthium* aqueous extract—A comprehensive study. Mater. Sci. Eng. C.

[55] Rastogi L., Arunachalam J. (2011). Sunlight based irradiation strategy for rapid green synthesis of highly stable silver nanoparticles using aqueous garlic (*Allium sativum*) extract and their antibacterial potential. Mater. Chem. Phys..

[56] Roddu A.K., Wahab A.W., Ahmad A., Taba P. Green-route synthesis and characterization of the silver nanoparticles resulted by bio-reduction process. Proceedings of the 3rd International Conference on Science (ICOS 2019).

[57] Rawat V., Sharma A., Bhatt V.P., Singh R.P., Maurya I.K. (2020). Sunlight mediated green synthesis of silver nanoparticles using Polygonatum graminifolium leaf extract and their antibacterial activity. Mater. Today Proc..

[58] Rizwana H., Alwhibi M.S., Al-Judaie R.A., Aldehaish H.A., Alsaggabi N.S. (2022). Sunlight-mediated green synthesis of silver nanoparticles using the berries of *Ribes rubrum* (red currants): Characterisation and evaluation of their antifungal and antibacterial activities. Molecules.

[59] Fletcher J., Bender C., Budowle B., Cobb W.T., Gold S.E., Ishimaru C.A., Luster S., Melcher U., Scherm H., Seem R.C. (2006). Plant pathogen forensics: Capabilities, needs, and recommendations. Microbiol. Mol. Biol. Rev..

[60] Huang C.H., Vallad G.E., Adkison H., Summers C., Margenthaler E., Schneider C., Hong J., Jones J.B., Ong K., Norman D.J. (2013). A novel *Xanthomonas* sp. causes bacterial spot of rose (*Rosa* spp.). Plant Dis..

[61] Rudramurthy G.R., Swamy M.K., Sinniah U.R., Ghasemzadeh A. (2016). Nanoparticles: Alternatives against drug-resistant pathogenic microbes. Molecules.

[62] Burdușel A.C., Gherasim O., Grumezescu A.M., Mogoantă L., Ficai A., Andronescu E. (2018). Biomedical applications of silver nanoparticles: An up-to-date overview. Nanomaterials.

[63] Liao C., Li Y., Tjong S.C. (2019). Bactericidal and cytotoxic properties of silver nanoparticles. Int. J. Mol. Sci..

[64] Sarmast M.K., Salehi H. (2016). Silver nanoparticles: An influential element in plant nanobiotechnology. Mol. Biotechnol..

[65] Jayalakshmi B., Raveesha K.A., Amruthesh K.N. (2021). Isolation and characterization of bioactive compounds from *Euphorbia cotinifolia*. Future J. Pharm. Sci..

[66] Awaad A.S., Alothman M.R., Zain Y.M., Zain G.M., Alqasoumi S.I., Hassan D.A. (2017). Comparative nutritional value and antimicrobial activities between three *Euphorbia* species growing in Saudi Arabia. Saudi Pharm. J..

[67] Voukeng I.K., Beng V.P., Kuete V. (2017). Multidrug resistant bacteria are sensitive to *Euphorbia prostrata* and six others Cameroonian medicinal plants extracts. BMC Res. Notes.

[68] Li H., Yang P., Zhang E.H., Kong L.M., Meng C.Y. (2021). Antimicrobial ent-abietane-type diterpenoids from the roots of *Euphorbia wallichii*. J. Asian Nat. Prod. Res..

[69] Pochapski M.T., Fosquiera E.C., Esmerino L.A., Dos Santos E.B., Farago P.V., Santos F.A., Groppo F.C. (2011). Phytochemical screening, antioxidant, and antimicrobial activities of the crude leaves’ extract from *Ipomoea batatas* (L.) Lam. Phacog. Mag..

[70] Dada A.O., Inyinbor A.A., Idu E.I., Bello O.M., Oluyori A.P., Adelani-Akande T.A., Dada O. (2018). Effect of operational parameters, characterization and antibacterial studies of green synthesis of silver nanoparticles using *Tithonia diversifolia*. PeerJ.

[71] Ovais M., Ayaz M., Khalil A.T., Shah S.A., Jan M.S., Raza A., Shinwari Z.K. (2018). HPLC-DAD finger printing, antioxidant, cholinesterase, and α-glucosidase inhibitory potentials of a novel plant *Olax nana*. BMC Complement. Alter. Med..

[72] Chahardoli A., Karimi N., Fattahi A. (2018). *Nigella arvensis* leaf extract mediated green synthesis of silver nanoparticles: Their characteristic properties and biological efficacy. Adv. Powder Technol..

[73] Santos J., Oliveira M.B.P.P., Ibáñez E., Herrero M. (2014). Phenolic profile evolution of different ready-to-eat baby-leaf vegetables during storage. J. Chromatogr. A.

[74] Zahoor A., Munir H., Alam M.O., Ali R., Mahmood H., Usmanghani K., Husssain S. (2017). Quantitative HPLC Analysis of Vitamin D3 and Gallic Acid in Vivabon syrupfor Children Growth. RADS J. Pharm. Pharm. Sci..

[75] Zahoor M., Shah A.B., Gul S., Amin S. (2018). HPLC-UV analysis of antioxidants in *Citrus sinensis* stem and root extracts. J. Chem. Soc. Pak..

[76] Dada O.A., Adekola F.A., Odebunmi E.O. (2016). Kinetics and equilibrium models for sorption of Cu (II) onto a novel manganese nano-adsorbent. J. Dispers. Sci. Technol..

[77] Kumar B., Vizuete K.S., Sharma V., Debut A., Cumbal L. (2019). Ecofriendly synthesis of monodispersed silver nanoparticles using *Andean Mortiño* berry as reductant and its photocatalytic activity. Vacuum.

[78] Tippayawat P., Phromviyo N., Boueroy P., Chompoosor A. (2016). Green synthesis of silver nanoparticles in *aloe vera* plant extract prepared by a hydrothermal method and their synergistic antibacterial activity. PeerJ.

[79] Aziz N., Faraz M., Sherwani M.A., Fatma T., Prasad R. (2019). Illuminating the anticancerous efficacy of a new fungal chassis for silver nanoparticle synthesis. Front. Chem..

[80] Bhuyan T., Mishra K., Khanuja M., Prasad R., Varma A. (2015). Biosynthesis of zinc oxide nanoparticles from *Azadirachta indica* for antibacterial and photocatalytic applications. Mater. Sci. Semicond. Process..

[81] Masum M., Islam M., Siddiqa M., Ali K.A., Zhang Y., Abdallah Y., Li B. (2019). Biogenic synthesis of silver nanoparticles using *Phyllanthus emblica* fruit extract and its inhibitory action against the pathogen *Acidovorax oryzae* strain RS-2 of rice bacterial brown stripe. Front. Microbiol..

[82] Singh P., Mijakovic I. (2022). Antibacterial Effect of Silver Nanoparticles Is Stronger If the Production Host and the Targeted Pathogen Are Closely Related. Biomedicines.

[83] Yin I.X., Zhang J., Zhao I.S., Mei M.L., Li Q., Chu C.H. (2020). The antibacterial mechanism of silver nanoparticles and its application in dentistry. Int. J. Nanomed..

[84] Ghabban H., Alnomasy S.F., Almohammed H., Al Idriss O.M., Rabea S., Eltahir Y. (2022). Antibacterial, Cytotoxic, and Cellular Mechanisms of Green Synthesized Silver Nanoparticles against Some Cariogenic Bacteria (*Streptococcus mutans* and *Actinomyces viscosus*). J. Nanomater..

[85] Pohanka M. (2016). Toxicology and the biological role of methanol and ethanol: Current view. Biomed. Pap. Med. Fac. Palacky Univ. Olomouc.

[86] Yousefi M., Afshari R., Sadeghi M., Salari R. (2018). Measurement of methanol and ethanol contents in most commonly used herbal distillates produced by three famous brands. Iran. J. Public Health.

